# Supplementary material for: Intention to Catha edulis chewing cessation and associated factors among Catha edulis chewers of Bahir Dar University students, Northwest Ethiopia: Application of the Trans theoretical model
Source: PLOS Glob Public Health. 2025 Jun 9;5(6):e0004525. doi: 10.1371/journal.pgph.0004525 (PMC12148129; doi:10.1371/journal.pgph.0004525)
Supplement: S1 Text — (DOCX) [file pgph.0004525.s001.docx]

**English questionnaire**

**Part I. Socio-demographic Characteristics of the participants**

| **Circle the number that indicates how well each of the following statements describes you for choices and fill the blank space with a relevant word or phrase** | | |
| --- | --- | --- |
| Q.No | Question | Choices |
| 101 | What is your Sex? | 1= Male 2=Female |
| 102 | How old are you? | ______________ years |
| 103 | What is your religion? | 1=Orthodox 2=Muslim 3=Protestant 4=Catholic 5=Other (specify) ________ |
| 104 | What is your year of study? | 1=first year 2= second year 3=third year 4= fourth year 5=fifth year 6=six year |
| 105 | What is your department? | Specify your department______________________ |
| 106 | How much is your Monthly pocket money? | __________ birr |

**Part II. Khat chewing history of participants**

| **Circle the number that indicates how well each of the statements describes you for choices and fill the blank space with a relevant word or phrase.** | | |  |
| --- | --- | --- | --- |
| 201 | What was your age when you started chewing khat? | _____________ years |  |
| 202 | For how many years you have chewed khat? | _____________ years |  |
| 203 | During the past 30 days, how many days did you chew khat? | _____________ days |  |
| 204 | How frequently do you usually chew khat per week? | 1 = sometimes 2 = 1-3 days per week  3 = 4-6 days per week 4 = day to day |  |
| 205 | For how long do you usually Chew khat within 24 hours? | ____________ hours |  |
| 206 | What is the Estimate of daily khat consumption in birr? | ____________ birr |  |
| 207 | Why do you chew khat currently? | 1=Reading 2=Pray 3=Recreation 4= Other __________ |  |
| 208 | With whom do you usually chew khat? | 1=Friends 2=Family 3=Alone 4= Other __________ |  |
| 209 | Additional substance use with khat if any? | 1=alcohol 2=cigarette 3=hashish 4=other ________ 5= none | If none go to Q. 211 |
| 210 | What is the usual frequency of additional substance use? | 1 = sometimes 2 = 1-3 days per week  3 = 4-6 days per week 4 = day to day |  |
| 211 | What do you think the barriers are if khat chewers want to stop khat chewing? | 1= peer pressure 3= negative Emotion  2= withdrawal effect 4= others (specify) _______________ |  |

**Part III. Participant’s intention/stages of change/ to stop khat chewing**

| **Circle the number that indicates how well each of the following statements describes your feeling**. | | |  |
| --- | --- | --- | --- |
| 301 | Are you seriously considering quitting khat chewing within the next 6 months? | 1=Yes 2=No | If No go to Q.401 |
| 302 | Are you planning to quit khat chawing within 30 days? | 1=Yes 2=No | If No go to Q. 401 |
| 303 | Have you had 24 hours of quite attempts experience in the past year? | 1=Yes 2= No |  |

**Part IV. Participant Process of changes to stop khat chewing**

| **Circle the number that describes your thought (1=Strongly Disagree 2= Disagree 3=Neutral 4= Agree 5= Strongly Agree)** | | |
| --- | --- | --- |
| **Questions to measure Consciousness Raising** | | |
| 401 | I read a book or an article about the problems of khat chewing | 1 2 3 4 5 |
| 402 | I listened to experts’ explanations about the effect of khat chewing. | 1 2 3 4 5 |
| 403 | I watched advertisements on how to stop chewing. | 1 2 3 4 5 |
| 404 | I recall information people had given me to stop khat chewing | 1 2 3 4 5 |
| **Questions to measure Dramatic Relief** | | |
| 405 | I react emotionally to warnings about chewing khat. | 1 2 3 4 5 |
| 406 | Warnings about the health hazards of khat chewing move me emotionally. | 1 2 3 4 5 |
| 407 | Remembering studies about illnesses caused by chewing upsets me. | 1 2 3 4 5 |
| 408 | Dramatic portrayals of the evils of chewing affect me emotionally. | 1 2 3 4 5 |
| **Questions to measure Environmental Reevaluation** | | |
| 409 | I am considering the belief that people quitting khat chewing will help to improve the world | 1 2 3 4 5 |
| 410 | I am considering the idea that the world could be a better place without my chewing | 1 2 3 4 5 |
| 411 | I think that chewing is polluting the environment. | 1 2 3 4 5 |
| 412 | I reassess the fact that being content with myself includes changing the chewing habit. | 1 2 3 4 5 |
| **Questions to measure Self Reevaluation** | | |
| 413 | I pondered on my roles as a family member while I was a khat chewer. | 1 2 3 4 5 |
| 414 | I consciously struggle that khat chewing contradicts my view of myself as a caring and responsible person | 1 2 3 4 5 |
| 415 | I get upset when I think about my khat-chewing habit | 1 2 3 4 5 |
| 416 | My dependency on khat makes me feel disappointed in myself | 1 2 3 4 5 |
| **Questions to measure Social Liberation** | | |
| 417 | I Notice that public places have sections set aside for khat chewing. | 1 2 3 4 5 |
| 418 | I Find society changing in ways that make it easier for the non-chewers. | 1 2 3 4 5 |
| 419 | I notice that nonchewers are asserting their rights | 1 2 3 4 5 |
| 420 | I am aware of more and more people encouraging me to quit khat chewing. | 1 2 3 4 5 |

**Part V. Participant Self Efficacy**

| **If you are engaged in a quitting attempt, how confident are you to refrain from chewing khat?**  **(1 = Not Very Confident 2 =Not Confident 3 = Neutral 4 = Confident 5 = Very Confident)** | | |
| --- | --- | --- |
| 501 | When things are just not going the way, I want and I am frustrated | 1 2 3 4 5 |
| 502 | With my close friends who is chewing | 1 2 3 4 5 |
| 503 | When there are arguments and conflicts with my friends. | 1 2 3 4 5 |
| 504 | When I am happy and pass my exam. | 1 2 3 4 5 |
| 505 | When I failed the exam. | 1 2 3 4 5 |
| 506 | When I would experience an emotional crisis. Such as an accident or death in the family. | 1 2 3 4 5 |
| 507 | When I see someone chewing and enjoining it. | 1 2 3 4 5 |
| 508 | Over coffee while talking and relaxing. | 1 2 3 4 5 |
| 509 | When I realize that quitting khat chewing is an extremely difficult task for me. | 1 2 3 4 5 |
| 510 | When I am craving a khat. | 1 2 3 4 5 |
| 511 | When I begin to let down on my concern about my health and I am less physically active | 1 2 3 4 5 |
| 512 | When I wake up in the morning and face a tough day. | 1 2 3 4 5 |
| 513 | When I am extremely depressed. | 1 2 3 4 5 |
| 514 | When I am extremely anxious and stressed. | 1 2 3 4 5 |

**Part VI. Participants Decisional Balance**

| **Circle the number that indicates how well each of the following statements describes you (1= Strongly Disagree 2= Disagree 3= Neutral 4= Agree 5= Strongly agree)** | | |
| --- | --- | --- |
| **Questions to measure the Pros of Khat chewing** | | |
| 601 | Khat chewing relief tension. | 1 2 3 4 5 |
| 602 | Khat chewing helps me concentrate and do my exam better. | 1 2 3 4 5 |
| 603 | I am relaxed therefore more pleasant when chewing. | 1 2 3 4 5 |
| 604 | I like myself better when I chew. | 1 2 3 4 5 |
| 605 | If I try to stop chewing, I will be irritable and a pain to be around. | 1 2 3 4 5 |
| 606 | By continuing to chew, I feel I am making my own decisions. | 1 2 3 4 5 |
| **Questions to measure Cons of khat chewing** | | |
| 607 | I am embarrassed to have chew khat. | 1 2 3 4 5 |
| 608 | My chewing affects the health of others. | 1 2 3 4 5 |
| 609 | Others close to me would suffer if I became ill from chewing khat. | 1 2 3 4 5 |
| 610 | People close to me disapprove of my chewing. | 1 2 3 4 5 |
| 611 | My chewing khat bothers other people. | 1 2 3 4 5 |
| 612 | Chewing khat is hazardous to my health | 1 2 3 4 5 |

**Part VII: Khat dependency scale**

| **Circle the number for each statement that best describes you (1=Exactly Not True 2=Not True 3=Neutral 4=True 5=Exactly true**) | | |
| --- | --- | --- |
| 701 | If not chew for a while I feel restlessness and irritability | 1 2 3 4 5 |
| 702 | Whenever I go without a chew for a few hours, I experience a craving | 1 2 3 4 5 |
| 703 | When I am really craving a khat feels like I am in the grip of some unknown force that I cannot control | 1 2 3 4 5 |
| 704 | I would rather not travel by bus if I wouldn’t be allowed to chew | 1 2 3 4 5 |
| 705 | Sometimes, without realizing it I go for several hours or more without chewing. | 1 2 3 4 5 |
| 706 | I chew just about the same amount of khat from day to today | 1 2 3 4 5 |
| 707 | If I wake up during the night, I feel I need a khat | 1 2 3 4 5 |
| 708 | I can function much better in the morning after I have had a khat. | 1 2 3 4 5 |
| 709 | I feel a sense of control over my chewing, I can take it or leave it at any time | 1 2 3 4 5 |
| 710 | Whenever I quit or cut down on chewing, it is an unpleasant experience | 1 2 3 4 5 |

**Thank You**
