# Supplementary material for: Intention to Catha edulis chewing cessation and associated factors among Catha edulis chewers of Bahir Dar University students, Northwest Ethiopia: Application of the Trans theoretical model
Source: PLOS Glob Public Health. 2025 Jun 9;5(6):e0004525. doi: 10.1371/journal.pgph.0004525 (PMC12148129; doi:10.1371/journal.pgph.0004525)
Supplement: Attachment — Submitted filename: Response to Reviewers.docx [file pgph.0004525.s003.docx]

**Date: March 28, 2025**

**Rebuttal Letter**

PGPH-D-24-02892

Intention to Khat Chewing Cessation and Associated Factors Among Khat Chewers of Bahir Dar University Students; Application of Trans Theoretical Model

PLOS Global Public Health

**Zeamanuel Anteneh Yigzaw**

**To PLOS Global Public Health**

**Dear all,**

We the authors of this manuscript are pleased to thank the journal editors and the reviewers for revising the manuscript and giving your valuable and constructive comments and suggestions that help to improve the manuscript. We have made a rigorous revision of the manuscript as per your questions and comments. We have included the point-by-point response in the table below framed as editors’ comments/questions and authors’ responses. **We have made a severe revision on the entire manuscript that we believe had merit in improving the manuscript and attached it as tracked change and clean version separately**. We are happy to receive additional revisions if any that would have merit in improving the manuscript.

| **Review Comments to the Author** | **Authors Response** |
| --- | --- |
| **Reviewer 1 - General Comments:** |  |
| - Several lines of evidence portray the transtheoretical model (TTM) as the dominant model for health behavior change. The TTM is not only based on the critical assumptions about the nature of behavior change but also on interventions that can best facilitate such change. | **Thank you**  Transtheoretical Model (TTM) is not the only dominant model for health behavior change, although it is a prominent and widely used framework. Other models, such as the Health Belief Model (HBM) and Social Cognitive Theory (SCT), are also influential in understanding and promoting health behavior change.  Therefore, to know the stages of behavior change TTM is best model than among others. That is why we use TTM for this study. |
| - We have a number of published reports from Ethiopia (Dessie, Gondar, and Dire Dawa) on assessing the intention to stop khat chewing as the authors alluded in their text. What additional value the present study can possibly add to the existing body of knowledge in the area? The work could have added value had it included data on interaction among the different core constructs. For example, the interaction between the stages and decision balance. One could look at whether the pros of changing are higher/lower than the cons for subjects in the precontemplation/contemplation stage. | **Thank you**  Although there were written policies, laws, rules, and regulations to limit and prevent khat chewing, these were not enough to bring behavioral changes. Therefore, stage-based behavioral theory and models were essential to develop effective interventions for university students. Even though a number of studies on khat chewing were conducted, most studies do not well investigate university students’ khat chewing cessation intention through trans theoretical model.  Therefore, this study provides information at which stages khat chewer university students are they and construct stage-based interventions. It also provides insight to health researchers for further investigations on university students' intentions to khat chewing cessation. It also provides information’s for policymakers to create effective interventions at various levels of behavioral change stages. |
| **Title:** • The accepted scientific name for khat is Catha edulis (vahl) forssk. ex endl. (celestraceae). Please use this name in the title and when you first mention the plant in the Abstract and the text. | **Thank you**  we wrote the name khat as Catha edulis based on the direction given, word by word in the whole document. |
| **Introduction:** • The Introduction is bulky and should be significantly slashed. Introduction is needed to set the contacts and frame readers expectations. Thus, the authors need to focus on issues that are relevant to the present study. Please get some help to improve the language use. For example, Line 100: in the “horn” not the “horns’ of Africa. Line 108: khat “is” not “was” ingested… Line 188: Hawassa or Bahir Dar University?? | **Thank you**  Amended based on the recommendation given, now the introduction part was too short and the contents were also related only to the outcome variable precisely. |
| **Methods:** • I presume the questionnaire are adopted/adapted from TTM. Were they translated to the local language? If translated, were there any back translation effort to ensure consistency of the translation? If translated/back translated, who did the | **Thank you**  Yes, the questionnaire was adapted from different literatures, considering TTM as a frame.  The questionnaire was translated to Amharic by a language translator to keep consistency and for data quality. |
| • Were there no undecided participants in evaluation of the decisional balance?? | **Thank you**  Yes, we include undecided participants for decisional balance, moreover we ask them on pros and cons of cessation of khat chewing. |
| **Results:** • In the association studies, it is not clear which factors are included in the bivariate and multivariate analysis. Did the authors include variables listed in Table 3 (for e.g., age at which chewing started and use of additional substances) in the regression analysis? | **Thank you**  We include all variables for bivariate analysis which fill cross tabulation and we include factors which were significant at bivariate for multivariate analysis and finally we get five significant variables. |
| • As Table 5 stands now, the interpretation refers to the column “no” than to “yes”. For example, the interpretation “khat chewers with high consciousness-raising scores were five times more likely to have khat chewing cessation intention than those with low consciousness-raising [AOR=4.58 (2.43, 8.66)]” is not correct. To interpret as such, the authors need to switch the “no” and “yes” column. The first column in the intention to cease khat should be the “yes” column. | Thank you  We rewrite and restructure the table based on the comments given. |
| Discussion and Conclusion: • Any limitations of the study? Probably related to the design, the model used, or bias (for example, social desirability). | Thank you  We included the strength and limitations of the study based on the comments given. |
| **Reviewer 2 -** |  |
| **Abstract:** • The abstract needs revision. The background is shallow; the results should be presented in a meaningful manner than presenting the effect sizes only. The conclusion should give the implications than presenting comparisons. | Thank you  We rewrite the abstract based on the comment given. |
| **Methods:** • Terms such as Consciousness-raising, Dramatic relief and Environmental re-evaluation need definition. You used 10 items of Likert scale question, and you have used binary logistic regression, but Likert scale is treated like a continuous variable, and it is not appropriate to dichotomize it while having a neutral in the middle. Hence the study will benefit from a liner regression model. | Thank you  We operationally define those variables based on the comments given see from 239 – 259 in the track change.  Moreover, we can logistic regression for Likert scale since we use dichotomous variable as an outcome variable that is intention and no intention. |
| • Sampling and selection: Need to show clearly the selection and allocation to levels of students as first year, second and so on and also departments. • How do you identify and select chewers? | **Thank you**  All six campuses of BDU students were 15038 during the study period, then distribution of all students to their respective campuses done, an estimated khat chewer students in all six campuses of BDU students was 1579. Allocation of khat chewer students to each BDU campus was done (Poly, peda, selam, zenzelema, yibab and sebatimit). Then allocation of study population to each campus was done. |
| **Results:** • Daily hours of chewing are stratified into three 1-3 hours, 4-6 hours and >6 hours- where more than 10% reported to chew for more than 6 hours, how believable is this data? Which year are these students? Please check this is too much time to take for a student | **Thank you**  Yes, it can be because 63.2% of students chew Catha edulis for the purpose of reading, since the main task of university students was reading it can be.  More over most of the students were above the second year that means senior students. |
| **Discussion:** • Can you give meanings to your findings than simply comparing your findings with others? | **Thank you**  We try to rephrase the sentence based on the comments given |
| **Conclusion:** • This is good may need some more work after revision of the statistics. | **Thank you**  We try to rephrase and make good based on the comments given. |
|  |  |

**Thank you very much**
