# Supplementary material for: Intention to Catha edulis chewing cessation and associated factors among Catha edulis chewers of Bahir Dar University students, Northwest Ethiopia: Application of the Trans theoretical model
Source: PLOS Glob Public Health. 2025 Jun 9;5(6):e0004525. doi: 10.1371/journal.pgph.0004525 (PMC12148129; doi:10.1371/journal.pgph.0004525)
Supplement: Attachment — Submitted filename: Response_to_Reviewers_auresp_2.docx [file pgph.0004525.s004.docx]

**Date: May 03, 2025**

**Rebuttal Letter**

PGPH-D-24-02892R1

Intention to Khat Chewing Cessation and Associated Factors Among Khat Chewers of Bahir Dar University Students; Application of the Trans Theoretical Model

PLOS Global Public Health

**Zeamanuel Anteneh Yigzaw**

**To PLOS Global Public Health**

**Dear all,**

We, the authors of this manuscript, are pleased to thank the journal editors and the reviewers for revising the manuscript and giving your valuable and constructive comments and suggestions that helped to improve the manuscript. We have made a rigorous revision of the manuscript as per your questions and comments. We have included the point-by-point response in the table below framed as editors’ comments/questions and authors’ responses. **We have made a severe revision on the entire manuscript that we believe had merit in improving the manuscript and attached it as tracked change and clean version separately**. We are happy to receive additional revisions if any that would have merit in improving the manuscript.

| **Review Comments to the Author** | **Authors Response** |
| --- | --- |
| **Reviewer 1** |  |
| **Justification for the study:** The value the present study adds to the existing body of knowledge is rebutted by saying that the study was performed on university students while the others were in the community. The authors need to explain how the findings obtained from university students are different from those reported from the community. | **Thank you**  Khat chewing among university students was different from the community, because university students chew khat for increasing their cognitive ability / for academic activity, performance especially during exams/, due to this it is unique among others, while most of the community chew khat due to addiction, religious issue or recreation or any other reason. Moreover, even though few studies have been conducted on khat chewing in Ethiopia, there is sufficient information in our study area, especially on university students. |
| **Analysis:** The authors need to look into the interaction among the different core constructs. For example, the interaction between the stages and decision balance. | **Thank you**  The interaction between the stages of change and decisional balance in the Transtheoretical Model (TTM) suggests that as individuals move through different stages of change, their perceptions of the pros and cons (decisional balance) of changing a behavior shift. Early stages (precontemplation, contemplation, preparation) are characterized by more cons perceived than pros, while later stages (action, maintenance) see a shift towards more pros perceived than cons. |
| **Reviewer 2:** |  |
| **General:** The MS has improved a lot but still needs some work for clarity before it is published. Also, editorial work is also important. | **Thank you**  We grammatically amend the whole document using standardized grammar checker software. |
| **Title:** Minor comment on the title: It is important that you show the country too | **Thank you**  Included as per the direction given. |
| **Abstract:** - **Background:** The statement “Over 10 million people chew Catha edulis worldwide". The student’s behavioral stage of change toward Catha edulis chewing cessation intention has not been thoroughly investigated”. This statement does not show anything about Ethiopia and also the word ‘students’ does not indicate which students. Place and population are important in abstracts as it is a concise version of the MS. | **Thank you**  We correct the manuscript as per the direction given, see lines 67 – 69. |
| - **Methods** the TTM is not indicated in the methods, in addition, the use an active voice is important for example the study used/ we used than a passive one. The language flow is a bit fragmented and needs some revision. | **Thank you**  We correct as per the direction given, see line 73. |
| - **Conclusion:** The conclusion could benefit if the implication of the study is indicated. Yes, intention is for cessation is low, but at what stage and also where should behavioral intervention should focus? | **Thank you**  Corrected as per the direction given, see lines 91 – 93 and lines 436 – 439. |
| **Background:** - Looking back at the background, there is no mention about the theoretical model ‘TTM’; the authors need to introduce the model and its application in different works. | **Thank you**  We correct it as per the direction given, see lines 118 -125 in the track change. |
| **Methods:** - Has improved a lot - but the MS will benefit from the below comments: - If the inclusion and exclusion criteria come after the sampling and selection | **Thank you**  Corrected as per the comment given, see lines 157 – 170. |
| - The selection is not clear. Needs a table indicating the campuses, departments and year of study. | **Thank you**  We insert Figure 1 as a sampling procedure, see 240 -245 in the track change, and see Figure 1 in the figures. |
| - The statement ‘The stratification variable considered departments of Catha edulis chewer students’ is not clear. Do you mean that you were targeting departments with high concentration of chewers? | **Thank you**  We used all Bahir Dar University all campuses and we collect information from Bahir Dar university main directorate about the average number of Khat chewer students in the university, and we used simple random sampling to select each Catha edulis chewers in each department.  See line 240 – 245 in the track change. |
| - Did the study request whether the students start chewing after they joined campus/university? This will answer/clarify questions related with year of chewing and mean age of chewers. | **Thank you**  We used Catha edulis chewer university students, who were active, that is, chewing currently. |
| - The authors indicated they used a Likert scale and then mentioned they used a logistic regression as the outcome is dichotomous…. fine. Another question here is how do you come to a category of high, low and medium? This needs clarification on the methods as it will answer your model use too. | Thank you  We used binary logistic regression because we dichotomized intention as high and low intention, as we put in the operational definition. |
| **Discussion:** - The first sentence in the first paragraph is not necessary - as it is mentioned in the previous sections. | **Thank you**  Corrected as per the comments given. |
| - The main findings should be described in the first paragraph without using statistics to give the reader a whole picture of what you want to say in the discussion. | **Thank you**  Corrected as per the comments given |
| - Strength and limitation should come after discussing the main findings so that the reader could read the material understanding especially the limitations. | **Thank you**  We write the strengths and limitations of the study after the discussion |
| Following this, you can discuss each in separate paragraph and give meaning to your findings rather than comparing immediately and giving reasons for the difference. You can just compare with studies having similar findings saying … others also found similar findings… then cite and exit the paragraph. | **Thank you**  We try to amend based on the comments given. |

**Thank you very much**
